# Decoherence, Anti-Decoherence, and Fisher Information

**DOI:** 10.3390/e23081035

**Published:** 2021-08-12

**Authors:** Andres M. Kowalski, Angelo Plastino

**Affiliations:** 1 Departamento de Física, Universidad Nacional de La Plata, La Plata B1900, Argentina; kowalski@fisica.unlp.edu.arstin; 2 Comisión de Investigaciones Científicas (CICPBA), La Plata B1900, Argentina; 3 Consejo Nacional de Investigaciones Científicas y Tecnológicas, (IFLP-CCT-CONICET)-C. C. 727, La Plata B1900, Argentina

**Keywords:** information geometry, Fisher information, semiclassical descriptions

## Abstract

In this work, we study quantum decoherence as reflected by the dynamics of a system that accounts for the interaction between matter and a given field. The process is described by an important information geometry tool: Fisher’s information measure (FIM). We find that it appropriately describes this concept, detecting salient details of the quantum–classical changeover (qcc). A good description of the qcc report can thus be obtained; in particular, a clear insight into the role that the uncertainty principle (UP) plays in the pertinent proceedings is presented. Plotting FIM versus a system’s motion invariant related to the UP, one can also visualize how *anti-decoherence* takes place, as opposed to the decoherence process studied in dozens of papers. In Fisher terms, the qcc can be seen as an order (quantum)–disorder (classical, including chaos) transition.

## 1. Introduction

The essential quantum decoherence concept arose in the early 1980s due to, among others, Zeh, Zurek, and Habib [1,2,3]. The emergence of the classical world in which we live from its quantum substratum has become a compelling issue that attracts much exciting work and intense, enlightening discussion. We revisit it here from the viewpoint of information geometry and one of its central subjects: Fisher information.

Information geometry is the study of statistical models (families of probability distributions) from a Riemannian geometric perspective. In this framework, a statistical model plays the role of a manifold. Each point on the manifold is a probability distribution from the model [4]. In [4], the author proposed Fisher information as a Riemannian metric on the statistical manifold [5]. Thus, Fisher’s information measure (FIM) plays an essential role in information geometry. Indeed, FIM is the protagonist of the present study. We begin our proceedings with a brief FIM-sketch.

Let us consider a continuous probability distribution function (PDF) f(x). Its associated Shannon information measure (entropy) *S* is [6]

(1) S[f]=−∫fln(f)dx,

an estimate of the “global nature”. It is not very sensitive to the strong *S*—changes taking place in a small-sized region. The opposite instance is that of the above-mentioned Fisher’s information measure (FIM) F [5], which measures the gradient content of the distribution *f*. Accordingly, it is quite sensitive even to tiny, localized perturbations. FIM can be written as [5]

(2) $$F\left[f\right]\;=\;\int \;\left[\frac{|\vec{\nabla }{f|}^{2}}{f}\right]\;dx,$$

and can be regarded (1) as an estimate of the ability to assess the value of a parameter, (2) as the amount of information that can be extracted from a set of measurements, and (3) as a measure of the disorder of a system or phenomenon [5,7]. Its most salient characteristic lies in its role in the so-called Cramer–Rao inequality (CRI). The Fisher information linked to translations of a one-dimensional observable *x* with corresponding probability density f(x) is [8]

(3) $$I_{x}=\int dx\;f\left(x\right)\;{\left(\frac{\partial \ln f(x)}{\partial x}\right)}^{2}\;dx,$$

which obeys the above-mentioned CRI

(4) $${\left(\Delta x\right)}^{2}\geq I_{x}^{-1}$$

involving the variance of the stochastic variable *x* [8]

(5) $${\left(\Delta x\right)}^{2}=\langle x^{2}\rangle -{\langle x\rangle }^{2}=\int dx\;f\left(x\right)\;x^{2}-{\left(\int dx\;f(x)\;x\right)}^{2}.$$

The gradient operator significantly influences the contribution of minute local *f*—variations to the value of FIM, meaning that FIM is called a “local” factor. Local sensitivity is useful in layouts in which their description appeals to a notion of “order” [8].

Consider $P=\{p_{i};\;i=1,\cdots ,N\}$ as a discrete probability distribution set for a system with *N* possible states. The problem of loss of information due to discretization has been studied in, for example, [9,10,11] and references therein. It entails the loss of FIM’s shift-invariance, which does not matter here. In our FIM case, we follow Ferri and coworkers [12] by writing

(6) $$F\left[P\right]\;=\frac{1}{4}\;\sum_{i=1}^{N-1}\;2\frac{{\left(p_{i+1}-p_{i}\right)}^{2}}{\left(p_{i+1}+p_{i}\right)}\;.$$

If our system lies in a rather ordered state, represented by a narrow probability distribution function (PDF), we face a Shannon entropy of S∼0 and a FIM of $F∼F_{max}$. On the other hand, in a very disordered state, one can consider an almost flat PDF and F∼0 [13].

## 2. Our Semi-Quantum Model

We consider a special bipartite system. It is the zeroth mode contribution of a strong external field to the production of charged meson pairs [14,15]. The Hamiltonian is

(7) $$\hat{H}\;=\;\frac{1}{2}\left(\;\frac{{\hat{p}}^{2}}{m_{q}}\;+\;\frac{{P_{A}}^{2}}{m_{cl}}\;+\;m_{q}\omega^{2}{\hat{x}}^{2}\;\right).$$

where (*i*) $\hat{x}$ and $\hat{p}$ are quantum operators, (*ii*) *A* and $P_{A}$ are classical canonical conjugate variables, and (*iii*) $\omega^{2}={\omega_{q}}^{2}+e^{2}A^{2}$ is an interaction term that introduces nonlinearity, with $\omega_{q}$ being the frequency and *e* the charge. $m_{q}$ and $m_{cl}$ are masses corresponding to the quantum and classical systems, respectively. Our Hamiltonian represents a system–environment model, where the environment is the classical subsystem [5]. This is different from the commonly used system–bath model, in which the bath typically consists of infinity degrees of freedom (DOF) to make the system decoherent. Here, there is a single classical DOF. For a fully quantum mechanical theory, an extra bath would be needed to make these DOFs classical. Thus, we warn the reader not to confuse the current model with a system–environment model.

It is shown in [15] that dealing with Equation (7) is tantamount to facing an autonomous system of nonlinear coupled equations:

(8) $$\begin{array}{c} \frac{d\langle {\hat{x}}^{2}\rangle }{dt}=\frac{\langle \hat{L}\rangle }{m_{q}}\;,\;\\ \frac{d\langle {\hat{p}}^{2}\rangle }{dt}=-m_{q}\;\omega^{2}\langle \hat{L}\rangle \;,\;\\ \frac{d\langle \hat{L}\rangle }{dt}=2\left(\frac{\langle {\hat{p}}^{2}\rangle }{m_{q}}-m_{q}\;\omega^{2}\langle {\hat{x}}^{2}\rangle \right)\;,\;\\ \frac{dA}{dt}=\frac{P_{A}}{m_{cl}}\;,\;\\ \frac{dP_{A}}{dt}=-e^{2}m_{q}\;A\langle {\hat{x}}^{2}\rangle \;. \end{array}$$

where $2\hat{L}=\hat{x}\hat{p}+\hat{p}\hat{x}$, involving the correlation operator $(\hat{x}\hat{p}+\hat{p}\hat{x})/2$. The system of Equation (8) is deduced from Ehrenfest’s relations [15]. To study the classical limit, we also consider the classical counterpart of Equation (7)

(9) $$H\;=\;\frac{1}{2}\left(\frac{p^{2}}{m_{q}}\;+\;\frac{{P_{A}}^{2}}{m_{cl}}\;+\;m_{q}\omega^{2}x^{2}\right),$$

where all the variables are classical. Consider now a new quantity *I*, a motion invariant described by the system of the previously introduced equations (Equation (8)) and related to the uncertainty principle

(10) $$I\;=\;\langle {\hat{x}}^{2}\rangle \langle {\hat{p}}^{2}\rangle -\frac{{\langle \hat{L}\rangle }^{2}}{4}\geq \frac{\hbar^{2}}{4}.$$

A classical computation of *I* yields $I=x^{2}p^{2}-L^{2}/4\equiv 0$.

Via Hamilton’s equations, one can find the classical counterpart of Equation (8), with equations that look identical in appearance to Equation (8) if one replaces quantum mean values with classical variables; that is, $\langle {\hat{x}}^{2}\rangle \Rightarrow x^{2}$, $\langle {\hat{p}}^{2}\rangle \Rightarrow p^{2}$ and $\langle \hat{L}\rangle \Rightarrow L=2xp$. One reaches the classical limit by letting I→0 or the quantity (“relative energy”),

(11) $$E_{r}\;=\;\frac{E}{I^{1/2}\omega_{q}}\to \infty ,$$

($E_{r}\geq 1$), where *E* is the total energy of the system. In the present work, we use suitable arbitrary units and fix

(12) $$\omega_{q}=1,\;\mathrm{in}\;\;\;\mathrm{arbitrary}\;\;\;\mathrm{frequency}\;\;\;\mathrm{units},$$

and in the pertinent accompanying units,

(13) $$E=0.6,\;m_{cl}=m_{q}=1,\;A=1.$$

The charge is also e=1 in suitable units. We vary 0<I<∞. A measure of the degree of convergence between classical and quantum results in the limit $E_{r}\to \infty$ of Equation (11) can be found in the norm N of the vector $\Delta u=u-u_{cl}$ [15],

(14) $$N_{\Delta u}=\left|u-u_{cl}\right|,$$

where the three components of vector $u=(\langle {\hat{x}}^{2}\rangle ,\langle {\hat{p}}^{2}\rangle ,\langle \hat{L}\rangle )$ are the “quantum” parts of the solution of the system defined by Equation (8) and $u_{cl}=\left(x^{2},p^{2},L\right)$ is its classical counterpart. For the classical counterpart of Equation (8), I=0 can be obtained.

This model was studied in detail by the authors of [15], who plotted diverse dynamical quantities as a function of $1<E_{r}<\infty$, depicting a typical decoherence process.

Three $E_{r}$-zones are clearly distinguished: (1) quantum, (2) transitional (semi-classical), and (3) classical. Thus, a decoherence process is delineated that, as explained below, can be described by the *I* values. An interesting feature of this *I*-described decoherence picture resides in the fact that, in some special *I*-sub-region, *chaos is always found*.The relative number of chaotic orbits (with respect to the total number of orbits) increases as the decoherence intensifies. The associated orbits display traits that cannot appropriately be described via the global measure shown in Equation (14). A local measure such as FIM is required as a substitute. This is why, in this paper, we focus on coherence generation rather than on decoherence processes and describe our results in terms of FIM versus *I* (replacing the $E_{r}$ description by an *I* description). To repeat, we wish to describe the classical–quantum transition in Fisher terms using the invariant *I* (see figures below). We observe that, at certain values of *I* that are appropriately given special symbolic names that are self-explicative, interesting decoherence changes can be found.

- At a low *I* value, $I=I_{P}=0.0325$, chaos emerges;
- At a still lower value, $I=I_{class}=7.75\exp\left(-4\right)\approx 0.001$, the classical zone delineates itself;
- For $I\leq I_{class}$, the classical zone applies;
- The transition region corresponds to $I_{class}\leq I\leq I_{P}$;
- For $I\geq I_{P}$, we reach the quantum zone.

## 3. How to Determine Our Underlying Probability Distribution

The model study referred to in the previous section, which is of a statistical nature, necessitates an appropriate probability distribution. We employed a standard approach that is widely used to determine the underlying probability distribution function *P* associated with a given dynamical system or time series (in our case here, an *I*-series). Several of these standard schemes can be found; see for instance [16,17,18,19,20,21,22]. We opted for the most recent method (the Bandt–Pompe ordinal-patterns methodology [22]). Our data points were entered into the Bandt–Pompe method to obtain the probability distribution according to the solutions of Equation (8). Solving these, we extracted the values of <$x^{2}$>, with one result for each different *I*-value. These <$x^{2}$>-values constituted a time-series. There are many techniques that permit the extraction of a probability distribution out of a given time-series. We employed the Bant–Pompe methodology for this purpose; see the above cited references for more details.

The probability distribution *P* is obtained once we fix the so-called embedding dimension *D* and the time delay τ [22]. We have previously applied this methodology in [23,24]) and refer the reader to these references for specific details on working with the approach.

The Bandt–Pompe method for the evaluation of a probability distribution *P* is based on the details of the attractor reconstruction procedure. A notable Bandt–Pompe result is a clear improvement in the performance of the information quantifiers obtained by employing the BP *P*—generating algorithm. One must to assume that enough data are available for a correct attractor reconstruction. The advantages of the Bandt–Pompe method reside in (a) its simplicity and thus (b) its extremely fast computation-process, (c) its robustness, and (d) its invariance with respect to nonlinear monotonous transformations. Further, it may be applied to any kind of time series (regular, chaotic, noisy, or reality-based). It is important to remark that calculations made with the Bandt–Pompe method are robust in the presence of observational and dynamical noise. Of course, the embedding dimension *D* plays an important role in the evaluation of the appropriate probability distribution, since *D* determines the number of accessible states D! indicating what length *M* of the time series is needed in order to obtain reliable statistics.

## 4. Results

**Figure 1:**
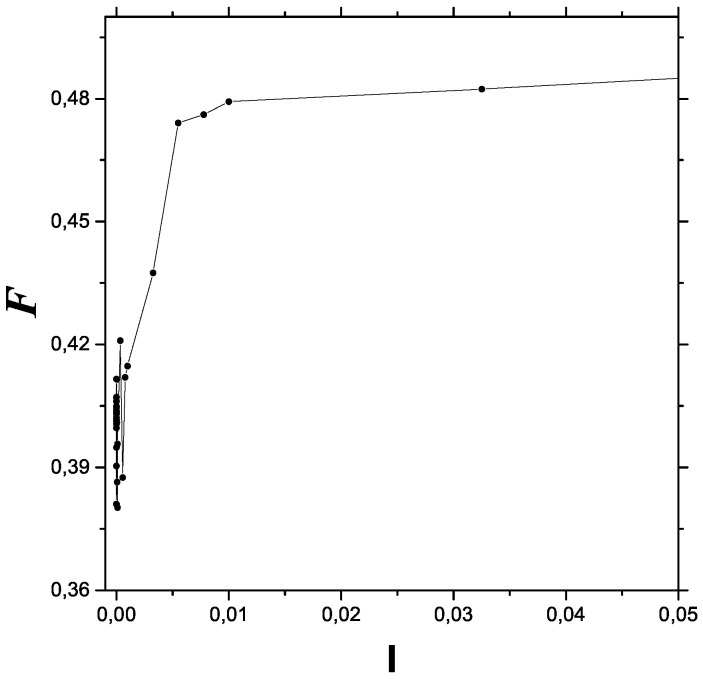
Anti-decoherence process. Fisher information F vs. *I* for an ample range that encompasses all three classical, semi-classical, and quantum regions.

Our data points as entered the Bandt–Pompe technique were obtained as the solutions of Equation (8). From them, we extracted the values of $\langle x^{2}\rangle$. We pass now to a description of them in terms of Fisher’s measure. For the initial conditions needed, we varied *I* to obtain our different Fisher values. Figure 1 depicts the results of FIM versus *I*. We considered 5000 data-points per initial condition and 41 different values of *I*. Pure classicality is seen at the extreme left, with a relatively low FIM value. FIM oscillations characterize the transition zone. Beyond this zone, an anti-decoherence process of growing FIM leads to the quantum zone on the right side. We see in Figure 1 rapid oscillations near the origin, the zone associated to classical chaos, which was proved to exist in this system in [15]. After this, the FIM grows, in an steadily ordering process, and then stabilizes itself as the maximum order-degree permitted by quantum uncertainty is reached.

**Figure 2:**
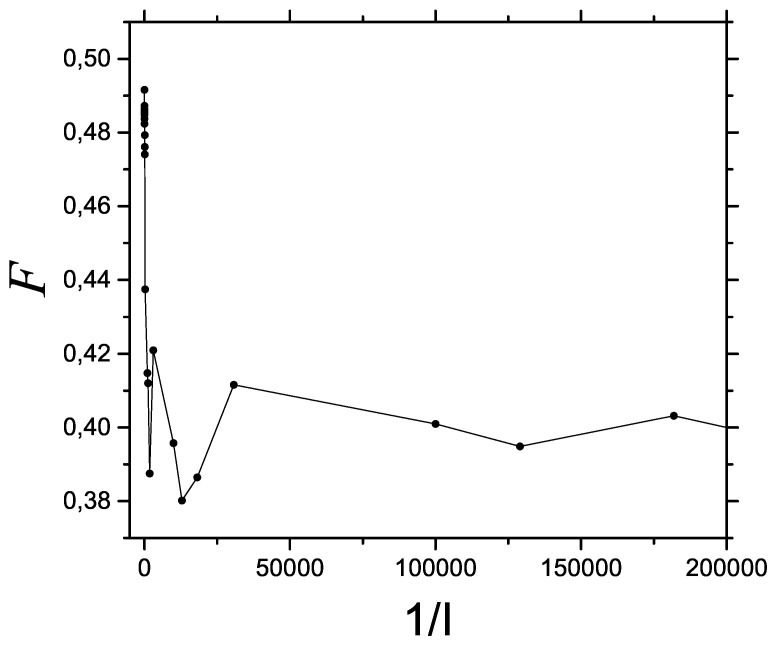
Decoherence process. Fisher information F vs. 1/I for an ample rater range that encompasses the three classical, semi-classical, and quantum regions. Classicity is seen on the right with a relatively high FIM value.

We pass now to Figure 2, which depicts a decoherence process. Before the decoherence process starts, FIM is higher in the quantum zone on the left side.

**Figure 3:**
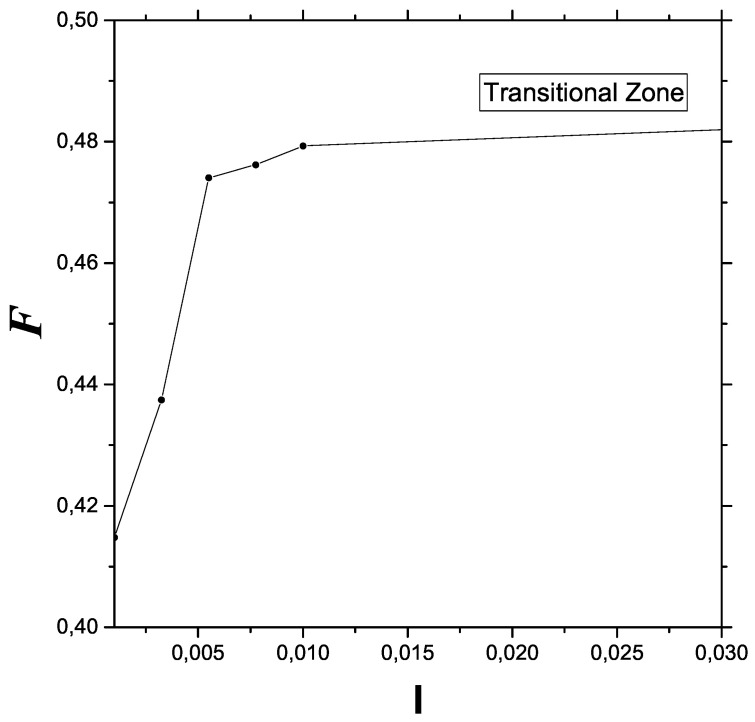
Details of the transitional semi-quantum zone are observable in this plot. In Fisher terms, the quantum–classical changeover can be seen as an order (quantum)–disorder (classical, including chaos) transition.

Plotting FIM versus *I* in an appropriate range allows one details of the transition zone to be observed in Figure 3 that are not easily available without the aid of Fisher information. The transition process consists of gaining information (represented by FOM) in a particular manner.

## 5. Conclusions

In this article, we have visualized the quantum–classical transition as a process of information gain (Fisher’s) when moving from the classical to the quantum region. In Fisher terms, the quantum–classical changeover can also be seen as an order (quantum)–disorder (classical, including chaos) transition.

This is because, in a fixed scenario (that of our physical model), one requires more information to compensate for quantum uncertainty in the quantum zone.

Our conclusions refer to the probability distribution that describes the classical–quantum transition in our model. This is of such a nature that its associated information quantifier *I* is an upper bound to the quantum uncertainty. FIM grows as the system anti-decoheres; that is, as it passes from the classical to the quantum realm. The latter’s description necessitates more information than the former. In the present work, we have studied the classical–quantum frontier problem by using the Fisher Information, considering the dynamics generated by a semi-classical Hamiltonian that represents the zeroth mode contribution of a strong external field to the production of charged meson pairs [14,15].

The features of the route from classicality to the quantum stage are depicted via (i) the motion invariant *I* and an upper bound to the quantum uncertainty, and (ii) by Fisher’s information measure (FIM). As *I* grows from zero (the “pure classical instance”) to finite values (the quantum situation), a significant series of *morphology changes* are exhibited. Our results are in complete accordance with those of [25,26,27].

## Data Availability

Data supporting reported results can be found here and in references [15,16,17].
